# WORKbiota: A Systematic Review about the Effects of Occupational Exposure on Microbiota and Workers’ Health

**DOI:** 10.3390/ijerph19031043

**Published:** 2022-01-18

**Authors:** Nicola Mucci, Eleonora Tommasi, Annarita Chiarelli, Lucrezia Ginevra Lulli, Veronica Traversini, Raymond Paul Galea, Giulio Arcangeli

**Affiliations:** 1 Department of Experimental and Clinical Medicine, University of Florence, 50139 Florence, Italy; nicola.mucci@unifi.it (N.M.); veronica.traversini@unifi.it (V.T.); giulio.arcangeli@unifi.it (G.A.); 2 Postgraduate Medical Training Programme in Cardiology, University of Perugia, 1 Piazza dell’Università, 06123 Perugia, Italy; eletommasi87@gmail.com; 3 Occupational Medicine Unit, Careggi University Hospital, 50134 Florence, Italy; chiarellian@aou-careggi.toscana.it; 4 Occupational Medicine School, University of Florence, 50139 Florence, Italy; 5 Faculty of Medicine & Surgery, University of Malta, MSD 2090 Msida, Malta; raymond.galea@um.edu.mt; 6 The Malta Postgraduate Medical Training Programme, Mater Dei Hospital Msida, MSD 2090 Msida, Malta

**Keywords:** microbiota, occupational health and safety, occupational medicine, occupational exposure, dysbiosis, host–microbe interaction

## Abstract

The characterization of human microbiota and the impact of its modifications on the health of individuals represent a current topic of great interest for the world scientific community. Scientific evidence is emerging regarding the role that microbiota has in the onset of important chronic illnesses. Since individuals spend most of their life at work, occupational exposures may have an impact on the organism’s microbiota. The purpose of this review is to explore the influence that different occupational exposures have on human microbiota in order to set a new basis for workers’ health protection and disease prevention. The literature search was performed in PubMed, Cochrane, and Scopus. A total of 5818 references emerged from the online search, and 31 articles were included in the systematic review (26 original articles and 5 reviews). Exposure to biological agents (in particular direct contact with animals) was the most occupational risk factor studied, and it was found involved in modifications of the microbiota of workers. Changes in microbiota were also found in workers exposed to chemical agents or subjected to work-related stress and altered dietary habits caused by specific microclimate characteristics or long trips. Two studies evaluated the role of microbiota changes on the development of occupational lung diseases. Occupational factors can interface with the biological rhythms of the bacteria of the microbiota and can contribute to its modifications and to the possible development of diseases. Future studies are needed to better understand the role of the microbiota and its connection with occupational exposure to promote projects for the prevention and protection of global health.

## 1. Introduction

The interest in the microbiota and its genetic heritage, microbiome, is a new growing field in medicine, and it is rapidly evolving. Each type of microbiota has its own influence on the psycho-physical state and well-being of organisms [1]. The recent development of sequencing techniques has made it possible to establish how much the microbiome is capable of conditional homeostasis and, therefore, of human health [2]. However, several issues need to be explained about the mechanisms through which the microbiota and its genetic heritage affect the body’s metabolic response. Human gut microbiota is composed of ∼100 trillion microorganisms, including over 500 genera of bacteria with two predominant phyla: *Bacteroidetes and Firmicutes* [3,4]. In the stomach and small digestive tract, relatively few species of bacteria are present. In the large intestine, most of the bacteria (99%) are anaerobes; however, in the cecum, high densities of aerobic microbes are recorded. The most dominant bacterial phyla in the human gut are *Firmicutes, Bacteroidetes, Actinobacteria*, and *Proteobacteria*, and the most recorded bacterial genera are *Bacteroides, Clostridium, Peptococcus, Bifidobacterium, Eubacterium, Ruminococcus, Faecalibacterium*, and *Peptostreptococcus* [5]. The nasal microbiome consists of *Actinobacteria*, *Firmicutes*, *Proteobacteria*, and the nasal vestibule is relatively enriched in *Firmicutes*, including *Staphylococcus aureus* [6]. On the skin, *Cutibacterium* and *Staphylococcus* species dominate sebaceous areas (such as the face and torso), while *Corynebacterium, Staphylococcus*, and *beta-Proteobacteria* are found in moist areas (such as the armpits and the elbow and knee creases) [7]. Recent studies suggest that a lower Firmicutes/Bacteroidetes ratio is linked with autoimmune diseases such as Systemic Lupus Erythematosus (LES) [8,9]. Similarly, recent studies show that microbiota’s alterations are playing an increasing role in the etiopathogenesis of many other metabolic and inflammatory diseases (obesity, diabetes, inflammatory bowel diseases, and asthma) as well as in neurological conditions (depression, anxiety, and Parkinson’s disease) [10,11,12,13,14,15]. Many factors can influence the composition of the microbiota. Smoking, for example, can decrease the amount of *Actinobacteria* and *Firmicutes* phyla as well as the genera *Bifidobacteria* and *Lactococcus* in microbiota but can determine an increase of *Proteobacteria* and *Bacteroidetes* [16]. Other factors can also alter the gut microbiota, such as age and diet [17,18]. Eating habits can cause fluctuations in the composition and function of the gut microbiota. If the rhythm of feeding is disrupted, then the microbiota’s diurnal rhythmicity in the gut is altered, and dysbiosis can occur [19,20]. As dietary nutrients have been proven to regulate the rhythm of the peripheral clock, emerging studies are showing that the gut microbiota may influence the body’s circadian rhythm and reprogram it [21,22]. In other words, there is a “gut-microbiota–circadian clock axis” according to which the circadian clock influences the composition of the gut microbiota, and conversely, the gut microbiota can also regulate the circadian rhythm, with evidence of bidirectional communication between the two [23,24,25]. The alteration of this axis is currently the focus of studies on changes in the microbiota due to shift work and jet lag [26]. Several recent works have also shown that gut microbiota plays an important role not only in the development of brain function but also in the pathology of stress-related diseases and neurodevelopmental disorders [27,28]. For example, even short-term exposure to stress can impact the microbiota community profile by altering the relative proportions of the main microbiota phyla. At the same time, experimental alterations of gut microbiota influence stress responsiveness, anxiety-like behaviors, and the neuroendocrine hypothalamic–pituitary–adrenal (HPA) stress axis [29,30,31]. The relationship between the human microbiota and occupational exposures is a field of increasing interest. Recent literature suggests that the microbiota may change in response to environmental exposures (e.g., to chemicals, metals, and particles) and that the microbiome may modulate itself the effect of these exposures [32]. Historically, the occupational sectors most studied have been those involving heavy exposure to potentially microbiome modifying factors, such as cotton textile factories and livestock farms [33,34,35]. The impact that occupational agents may have on workers’ microbiota is an emerging field with many social and global health implications. Further studies are needed to properly investigate the impact that the work environment, the occupational risks, and the types of jobs have on the composition of the worker’s microbiome. The main purpose of this literature search is to provide a systematic review of the existent studies regarding the relationship between occupational exposure and changes in the worker’s microbiota in order to identify appropriate protocols for risk prevention and workers’ health protection.

## 2. Materials and Methods

### 2.1. Literature Search

Our research aims at identifying original studies and reviews published in the last 20 years, from 2001 to 28 February 2021 on the major online databases: Pubmed database, Scopus, and Cochrane library. For this review, no protocol has been registered. The search string used was “(microbiome OR microbiota) AND (occupational*[TIAB] OR job*[TIAB] OR work*[TIAB])”. Our research strategy was conducted following the PICOs statement explained below. The population studied was that of workers with different exposures to factors that could influence their microbiota. In the studies selected, the intervention was represented by the collection and analysis of biological and environmental samples to characterize workers’ microbiota and its possible changes linked to job exposure. The outcome investigated was first the characterization of the basic microbiota of workers and subsequently the comparison with that of unexposed workers in order to carry out research on the microbiome and on the factors that can influence it. The revision process was carried out by two independent reviewers that read titles and abstracts identified by the search strategy. Duplicates were removed manually. Any doubts were solved through discussion with a third researcher experienced in the field. They selected the relevant articles in accordance with the inclusion and exclusion criteria, which are described below, in Section 2.2. Final eligibility was decided after carefully reading the full texts of the selected articles. The data required for the research were primarily obtained from published results but also integrated with online Supplementary Material when disposable (for example, details of the experiment performed or the methodology used to collect the biological samples). The authors manually extracted data from the selected studies. The useful data were collected in a spreadsheet, including the year of publication, the country of the workers surveyed, the number of participants, the type of workers, the tools used to collect the microbiota samples, and the results obtained in terms of microbiota variations.

### 2.2. Eligibility, Inclusion, and Exclusion Criteria

Studies assessing the relationship between occupational exposure and changes in the microbiota in workers were included in the study. All types of study designs were deemed eligible to be included in the review. No language or country restrictions were applied. Specifically, among the inclusion criteria we considered:

- Analysis of the baseline composition of the workers’ microbiota.
- Evaluation of comparisons between the microbiota of exposed workers and subjects not exposed to a particular occupational environment (animals included).
- Description of short- and long-term effects on the human microbiota due to occupational exposure.

The following exclusion criteria were respected for the selection of the articles:

- Studies conducted exclusively on animal models.
- Studies involving analysis of the microbiota in individuals not exposed to occupational risk factors or whose occupational exposure was not described in the preliminary recruitment phase of study participants.

### 2.3. Quality Assessment and Risk of Bias Assessment

Three different reviewers assessed the methodological quality of the selected studies with specific rating tools to assess the risk of bias in each study included (Table 1). We used the International Narrative Systematic Assessment (INSA) method to judge the quality of the narrative reviews [36] and the Newcastle Ottawa Scale (NOS) to evaluate cross-sectional, cohort studies, and case-control studies [37].

## 3. Results

**Figure 1:**
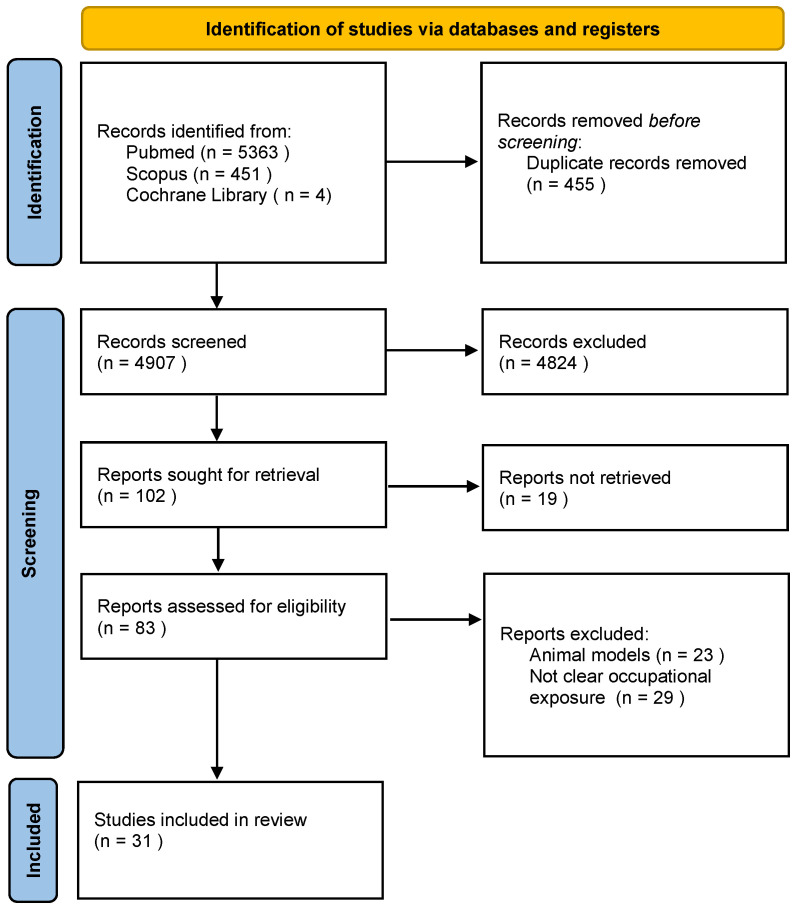
PRISMA 2020 Flow diagram for systematic reviews.

This systematic review follows the Prisma Statement [38]. The online research was performed on 15 February 2021, and it yielded 5818 studies: PubMed (5363), Scopus (451), and Cochrane Library (4). After the removal of duplicates, two authors independently screened the 4907 articles through title and abstract. Of these, 4824 were excluded because they did not include workers or occupational exposure. After this preliminary analysis, 102 reports were deemed eligible for retrieval. Any disagreement between the two authors about the inclusion of an article was solved through the intervention of a third researcher with expertise in the field. Of the 102 articles selected, the full text of 19 articles was not retrieved, and they were excluded from the review. After reading the full text of the 83 remaining articles, 52 were eliminated by applying the exclusion criteria by the two researchers. In total, 31 articles were finally included in our systematic review (Figure 1).

Of the 31 studies included in the systematic review, 4 were narrative reviews, 1 was a commentary, and 26 were original articles. Among the original articles, ten were cross-sectional studies, nine were longitudinal studies, six were case-control, and one was a cohort study (Table 2). The USA is the country in which most studies have been published (42.3%). The growing interest of the scientific community in studying the microbiome and the correlation with occupational exposure is demonstrated by the increasing trend of published studies highlighted by our review on the subject: one in 2009 (4%), four in 2017 (15.3%), eight in 2019 (30.7%), and as many as twelve in 2020 (46%). Among studies published in 2021, we included only one study because the literature search was performed in February 2021.

### 3.1. Original Articles

The scores assigned to the original articles have an average value of 6.3, a median of 6.5, and a modal of 7. These results suggest an average quality of the studies included in the review. A summary of the content of each article is included in the Supplementary Material, in Tables S1 and S2. The only study that scored full marks on the rating scale was a cohort study carried out in China in 2020 (NEW CASTLE 8).

#### 3.1.1. Tools for Microbiota Sampling and Analysis

The collection of biological material for the microbiota analysis was carried out using different techniques. The most common was fecal collection (10/26; 38.4%). In decreasing order of frequency, microbiota samples were collected through nasal swabs (9/26; 34.6%), oral swabs (6/26; 23%), skin samples (5/26; 19.2%), blood samples (3/26; 11.5%), nasopharyngeal swabs (2/26; 7.7%), and a swab of glove juice used by the worker (1/26; 3.8%). In eight studies (30.7%), the analysis was performed using two or more of the above-mentioned biological sampling methods. All biological sampling investigating the microbiota profile used the latest laboratory diagnostic methods, including PCR. In seven studies (27%), the collection of biological material from the worker was combined with sampling collected in the working environment. In all of these studies, air samples were collected. In almost half of the studies providing environmental monitoring, water analyses were also associated (3/7; 42.8%) (Table 3). PCR technique was also used in environmental samples to study the microbiome. In seven studies (27%), the use of health and lifestyle questionnaires was associated with the collection of the biological sample.

#### 3.1.2. Occupational Exposure and Workers’ Categories

The most studied occupational exposure was to biological agents (15/26; 57.7%). In particular, the most studied category of workers exposed to biological agents was farmers and butchers (12/15; 80%). Two studies (2/26; 7.7%) considered the effect of shift work on the workers’ microbiota. Exposure to chemical agents was also considered, and it was shown to alter the composition of the worker’s microbiota in four studies (4/26; 15.3%). Regarding occupational exposure to certain stressors and microclimate alterations, studies including workers subjected to long commutes and/or working in potentially unhealthy environments (e.g., military, sailors, diving divers, and tunnel workers) were included. The main occupational categories involved and their occupational exposures are summarized in Table 4. A summary of the main results divided by job category and professional exposure can be found in the following paragraphs.

#### 3.1.3. Works Involving Contact with Animals

Grant et al. found no statistically significant differences between the microbiota of workers in close contact with macaques and that of the animals tested. The analysis revealed differences between exposed workers’ microbiota and controls’ may be due to other factors (age, smoking status, and history of infectious diseases) [40]. Lai et al. also studied the impact on the human microbiome of working in an animal laboratory setting during a standard 8 h work shift. The change in the proportion of the workers’ microbiome when comparing post- vs. pre-shift samples did not reach statistical significance (*p* = 0.14 for the oral microbiome, *p* = 0.41 for the nasal microbiome, and *p* = 0.23 for the skin microbiome) [46]. Mbareche et al. demonstrated a statistically significant cluster between bacterial species (mainly *Firmicutes* and *Bacteroides*) found in air samples from swine barns and the nasopharyngeal flora of workers in contact with pigs, compared to a non-exposed control group (*p*-value of 0.0001) [49]. In addition, Kraemer et al. demonstrated a seasonal variability in the number of bacteria present in the air of pig barns (high values in winter compared to summer, *p* < 0.001) and that this fluctuation was reflected in dynamic changes in the nasal microbiota of exposed workers [45]. Wu et al., on the other hand, assessed the variation of the microbiota in exposed workers depending on whether the job was in more or less direct contact with the animal. In particular, bioaerosols from pig farms and nasal samples from pig farmers (at more contact with animals) had 31.7% shared OTUs (operational taxonomic units); more than those between pig slaughterhouses and slaughterhouse workers (23.4%) in which the contact with pigs was for less time (*p* < 0.001) [64]. A higher similarity in gut microbiome between farmers and swine than human non-exposed controls was found by Tan et al. Results from 16S-inferred fecal microbiota and metabolic profiles showed that only human control was significantly different from the swine with respect to farmers’ ones (*p*  < 0.05) [61]. Additionally, Sun et al. found that the swine farm environment can affect the fecal bacterial composition of exposed farmworkers. Farmworkers showed a less microbiota species diversity compared with the villagers and a greater similarity with swine’s gut profile (higher *Bacteroidetes* and *Clostridiaceae*, lower *Firmicutes*), suggesting a higher risk for their health [58]. To confirm these results, Sun et al. recently conducted a longitudinal investigation of swine farm environments’ impact on the gut microbiome of veterinary students who underwent occupational exposure during 3-month internships at swine farms. Multivariate analysis of OTUs composition revealed a modest yet significant change (R_2_ = 7.4%, permutational multivariate analysis of variance (PERMANOVA), *p* < 0.001) in students’ gut microbiotas that became more similar in composition to full-time farm workers’ gut microbiotas, and partially reverted 6 months after they returned home. Specific draft genomes rarely observed in the human gut shared 99.9 ± 0.1% (minimum 99.7%) 16S rRNA gene similarity and 99.5 ± 0.4% (minimum 98.9%) average nucleotide identity (ANI) between exposed students’ gut microbiome and environmental samples [59]. Similar results came out from the study of Islam et al. that investigated the composition and temporal dynamics of the nasal microbiome in people during and after long- and short-term exposure to livestock-associated MRSA clonal complex 398 (LA-MRSA CC398). All 221 pig farm workers’ nasal samples showed the presence of LA-MRSA CC398 (long-term exposure). Similarly, all samples collected immediately after the short-term exposure (temporary visitors) initially showed positivity for MRSA, whereas all 32 samples collected two hours before or 48 h after the visits were MRSA-negative [42]. Shukla et al. found maximum species richness in nasal microbiome dairy farmers (N_DF) compared with the ones from non-dairy farmers (N-NF, people that live and work in urban settings) (*p*-value < 0.0001). Additionally, the N_DF group had a lower burden of *Staphylococcus spp.* suggesting a correlation between higher microbial diversity and competition for colonization by staphylococci. The richness and higher microbial diversity in dairy farmers support the biodiversity hypothesis that living in urban environments could mean exposure to less diverse microbial flora with increased incidences of allergic and inflammatory diseases [56]. Peng et al. found significantly higher levels of *Proteobacteria* (*Pseudomonas and Acinetobacter*, *p* < 0.05) but lower *Actinobacteria* (*Corynebacterium* and *Propionibacterium*, *p* < 0.05) on forearm skin microbiota of farmworkers. Frequent farm animal operations also demonstrated a reduction in *Staphylococcus* and *Streptococcus* [51]. Only one study analyzed gender and age-related differences in animal-exposed workers’ microbiota. When stratifying by age category and livestock exposure, there were no OTUs significantly differentially abundant in those under 55 by livestock exposure. In those over 55, individuals with livestock exposure were significantly more likely to carry SR1 genera incertae sedis in their oropharynx (Log 2 -fold change: −23.17, adjusted *p*-value: <0.001) [43].

#### 3.1.4. Healthcare Workers

Healthcare workers are considered in three studies. An association between damaged hands from frequent washing in nurses and coagulase-negative staphylococci (*Staphylococcus haemolyticus*, *p* = 0.05) resulted in Rocha et al.’s analysis [54]. Rosenthal et al. found that washing hands >40 times per 12 h work shift in nurses had a reduced mean distance of the microbial communities indicating lower microbial pathogen community diversities [55]. Differences in the microbiota composition were linked to position role and workers’ department (ICU vs. non-ICU). Workers in the ICU showed a significant increase in the abundance of *Enterobacteriaceae, Pseudomonas*, and *Streptococcus* compared with non-ICU staff. Environmental samples resulted significantly closer to the medical workers’ microbiota respect to that of controls (*p* < 0.001) with the dominant environmental genus Pseudomonas that was more abundant in the gut microbiota of long-term than in the short-term workers’ [67].

#### 3.1.5. Metalworking Fluid Workers

Wu et al. conducted an invasive microbiota characterization—the only one described in or selection of articles—using lung biopsies in workers exposed to metalworking fluid (MWF) workers. MWF is a cooling and lubricating fluid that is frequently colonized by microorganisms such as *Pseudomonas*. Lung biopsies were conducted in MWF exposed symptomatic workers and showed the presence of characteristic MWF-bacteria species in a novel and distinct MWF-related pulmonary condition characterized by lymphocytic bronchiolitis and alveolar ductitis with B-cell follicles and emphysema (BADE) [70]. Wu also showed the presence of an OTU annotated to *Pseudomonas* (*Pseudomonas*_813945) in lung, skin, and nasal samples of exposed workers (Assembly and Machine Shop) as well as in MWF fluid. The sequence annotated as Pseudomonas_813945 in the 16S rRNA gene sequencing data most closely aligned with *P. andersonii, P. mendocina, P. pseudoalcaligenes,* and *P. oleovorans*. *Pseudomonas pseudoalcaligenes* was found in trace amounts in these samples (0.0000364%), and these reads could be perfectly matched (100% identity) to *P. pseudoalcaligenes* reads from metal working fluid samples, potentially suggesting that the bacterial DNA found in tissue samples might have originated from the metal working fluid. Interestingly, this OTU was not found differentially enriched in air samples suggesting that the main mode of transmission of this microbe is through contact rather than airborne [63].

#### 3.1.6. Workers Exposed to Dust

Two studies evaluated possible changes in the microbiome in workers exposed to dust (silica and ceramic dust). Zhou et al. analyzed gut microbiota characteristics in patients with early-stage pulmonary fibrosis due to silica exposure in the workplace. At the phylum level, *Firmicutes* and *Actinobacteria* abundances were lower in patients with silicosis (*p* < 0.05) with respect to healthy controls. This knowledge may be useful for the early diagnosis of silicosis and prevention of pulmonary fibrosis [69]. The composition of nasal microbiota of workers exposed to dust in ceramic factories was studied by Ahmed et al. Dust-exposed workers presented a significant increase in the relative abundance of phylum Proteobacteria, in particular *Haemophilus* spp. (*p* = 0.02), with a lower presence of *Actinobacteria* (*p* = 0.004) and *Bacteroidetes* (*p* = 0.01) with respect to controls [39].

#### 3.1.7. Workers Exposed to Pesticides

Only one study evaluated agricultural pesticide exposure-associated changes in the oral, buccal microbiota. Stanaway et al. found a seasonally persistent association between the detected blood concentration of the insecticide azinphos-methyl and the taxonomic composition of the buccal swab oral microbiome, in particular with significant reduction of *Streptococcus*. The persistence of this association from the spring/summer to the winter also suggests that long-lasting effects on the commensal microbiota have occurred [57].

#### 3.1.8. Shift Workers

Two studies investigated differences in the microbiome of shift workers. In night-shift workers, an increase of *Firmicutes* and *Actinobacteria* was found while *Bacteroidetes* showed a decrease. *Dorea longicatena* and *Dorea formicigenerans* were found to be significantly more abundant after the night shift (*p* = 0.005). *Faecalibacterium* abundance was found to be a biomarker of day shift work [50]. Swanson et al. showed that gut-derived short-chain fatty acids (SCFAs) in humans had diurnal rhythmicity and were impacted by night shift work [60].

#### 3.1.9. Military Personnel

Two studies involved military personnel. The upper respiratory microbiome of healthy military personnel in a garrison environment was studied by Hang et al. *Staphylococcus, Corynebacterium*, and *Propionibacterium* were more than 75% of all OTUs observed among the nasal and nasopharyngeal microbiota. *Streptococcus* was the only dominant bacterial genus (50% of all OTUs) in the oropharynx [41]. The second study investigated the relationship between microbiota variations in soldiers abroad and traveler’s diarrhea (TD) infection. *Ruminococcaceae* UCG-013 were found more abundant in TD+ subjects (possible susceptibility), while *Ruminiclostridium* sp. had higher relative abundance in TD- subjects (possible protection role). *Haemophilus* (*p*-value 0.0007) and *Turicibacter* sp (*p*-value 0.016) were shown to have a positive relationship with GI distress [62].

#### 3.1.10. Sailors

Zhang et al. explored the impact of the sea voyage on the intestinal microbiome of sailors. By comparing the intestinal microbiome of subjects at baseline (T0) and at the end of the sea voyage (T30), the analysis revealed an increase in the species *Streptococcus gordonii* and *Klebsiella pneumoniae* in sailors’ fecal samples [66].

#### 3.1.11. Tunnel Workers

Lu et al. conducted the only one study that correlated microbiota variations with altered mental status after exposure to stressful work conditions such as tunnel working environment. Tunnel workers after 3 weeks underground showed a gut microbial diversity significantly lower using the Shannon (*t* = 3.375, *p* = 0.001) and Simpson (*t* = 2.757, *p* = 0.008) indices respect to the baseline. After underground exposure, a higher abundance was found in the phylum *Actinobacteria*. The self-evaluation showed that at least one-half of the tunnel workers experienced one or more symptoms of mental distress (inattention, sleeplessness, loss of appetite, headache or dizziness, irritability) after working in the underground tunnel environment [48].

#### 3.1.12. Diving Sub-Sea Workers

One cross-sectional study evaluated the influence of commercial helium–oxygen saturation diving on divers’ gut microbiotas. Results showed a decrease of Bifidobacterium and of short-chain fatty acid (scFa)-producing bacteria (*Fusicatenibacter, Faecalibacterium,* and *Anaerostipes*) during and after diving activity. On the contrary, some pathogen species showed an increasing trend [71].

### 3.2. Reviews

Regarding the narrative reviews, the INSA score shows an average of 3.6, a median of 4, and a modal value of 5, thus indicating an intermediate quality of the studies. The most appropriate methodological reviews were conducted in the United States and China (INSA = 5). The content of each review article is summarized in Table S3 of the Supplementary Material.

A narrative review summarized evidence on the impact of the work microbiome and work-related chemical, metal, and particulate exposures on the human microbiome. Work-related environmental exposures are often orders of magnitude higher than in everyday life, spanning the spectrum of bioaerosols, chemicals, metals, and particles. Recent studies suggest that in particular work environments, such as strict contact with animals, the microbiome may change in response to exposures and that the microbiome may modulate the effect of these exposures. Work with animals appears to be associated with increased microbial diversity in the nasal microbiome of adult pigs and dairy farmers. Although limited, these studies support the idea that animal-related work influences the human microbiome. Even in a work setting where there is indirect animal exposure, the work microbiome may change the composition of the human nasal and skin microbiome. With regard to chemical compounds, arsenic has been shown to alter the characteristics of the workers’ microbiome [47]. Two narrative reviews and a commentary investigated the impact of altered circadian rhythms from shift work on the composition of the workers’ microbiome. A large number of clinical studies on shift workers and animal experiments have confirmed that circadian rhythm disorders play an important role in the pathogenesis of metabolic diseases. In particular, shift work is associated with increased risk for metabolic diseases, including type 2 diabetes, obesity, and metabolic syndrome. Recent research has shown that sleep and circadian disruption, via clock gene mutation or weekly shifts of the light-dark cycle, can negatively impact gastrointestinal tract function and produce dysbiosis [52]. Physiological and psychological stress have the capacity to disrupt the gut microbiota, negatively influence gut permeability, and contribute to poor health. Sleep loss and circadian misalignment are considered physiological stressors; a stressor–gut microbiota–inflammation–metabolic function pathway may explain the relationship between shift work and metabolic diseases [53]. It has been previously found that there are diurnal oscillations in the composition and function of gut microbiota; at the same time, the regulation of the gut microbiota is controlled by host feeding rhythms and influenced by the types of food consumed. If the rhythmic feeding times are disrupted, such as host genetic, molecular clock deficiency, and time-shift-induced jetlag, then aberrant gut microbiota diurnal rhythmicity and dysbiosis occur. Recent studies have shown that gut microbiota might be responsible for the reprogramming of circadian rhythmicity. A “gut–microbiota circadian clock axis” seems to exist with bidirectional communication between gut microbiota and the circadian clock. The microbiota gut–brain axis encompasses a bidirectional mode of communication between the microorganisms residing in our gut and our brain function and behavior. The composition of the gut microbiota is subject to diurnal variation and is entrained by host circadian rhythms. In turn, a diverse microbiota is essential for optimal regulation of host circadian pathways [68,72,73]. Additionally, significant relationships between nutrient intake and the circadian clock have been shown. Considering whether dysbiosis associated with sleep and circadian misalignment in shift workers affects clock genes in metabolic tissues will be important in future studies [68]. Only one narrative review searched for a link between exposure to occupational agents, changes in the microbiome, and possible development of health complications, in particular autoimmune disorders. Trichloroethene (TCE) exposure, which is known to induce/exacerbate Systemic Lupus Erythematosus in both experimental animals and humans, is also reported to cause alterations in the gut microbiome. In mice exposed to a high but occupationally relevant TCE dose, an increased abundance of genus Bifidobacterium and bacterial family Enterobacteriaceae was reported, along with a lower abundance of the genus Bacteroides and Lactobacillus, when comparing the exposed group to controls [44].

## 4. Discussion

The study of the “microbiota”, that is the characterization of the microorganisms present in the organism, and the analysis of the genetic components and metabolic functions of which this microbial community is capable (microbiome), is currently a topic of interest for the scientific community world [74]. The growing evidence on the impact that changes in the microbiota have on the etiopathogenesis of various diseases represents the main reason for this growing interest [75,76,77]. Understanding the mechanisms through which the microbiota can influence the development of various pathological conditions (gastrointestinal, cardiovascular, autoimmune) could, in fact, open new frontiers for the prevention, early diagnosis, and treatment. For this reason, the microbiota is currently being studied from the early stages of development of the individual up to adulthood with the attempt to identify factors involved in its modification (for example, dietary habits, lifestyles, consumption of fibers, and prebiotics) [78,79,80,81,82,83,84]. The goal is to understand if a risk factor, like occupational exposure, can cause a change in the composition of the microbiota, which can therefore be associated with the development of a particular pathology. The individual spends most of his life working, and for this reason, we hypothesized that even occupational exposure could contribute to changes in the organism’s microbiota. Our systematic review, therefore, aimed to identify studies that analyzed the baseline microbiota of workers exposed to certain risk factors and compare it with that of non-exposed individuals. An issue to consider when addressing the topic of the microbiome is the methodology used to collect and analyze samples. Each step of the microbiome study, from sample collection to storage, DNA extraction, and sequencing methods, can, in fact, influence the study results [85,86]. Our literature review confirmed the growing use of PCR gene amplification and sequencing of the specific 16S r RNA gene marker [87,88]. Our analysis revealed that collecting stool samples was the most used method to analyze workers ‘microbiota. Nevertheless, in several cases, the collection of different biological sources was reported (e.g., nasal samples, oral samples, skin samples, and blood samples). Several studies have also associated the analysis of the biological samples of workers with that of the environmental samples collected in the workplaces, aiming at better identifying the origin of specific changes in the microbiota. Exposure to biological risk was found to be the most studied occupational risk factor in our research on the microbiota. Most studies have shown changes in the composition of the microbiota of workers exposed to various forms of biohazard. Workers in contact with animals are the most studied. In particular, workers in close contact with farm animals (e.g., farmers) showed greater changes in the microbiota pattern than workers still exposed to biological risk at occupational level but with less direct contact with animals (e.g., slaughterers), suggesting a different profile risk based on the job performed. These results are in line with the study by Kraemer et al., who demonstrated an enormous impact of pig farming on the workers’ nasal microbiota and that the airborne microbiota of pig farms share heavy similarities with that of the workers’ nasal samples, suggesting that the environment influence the workers’ microbiota [89]. Improving the protection measures for the most exposed workers (for example, using masks) could reduce the impact of animal contact on the workers’ microbiota. Studies considering workers at less contact with animals, performing maintenance of the environments where the animals lived (e.g., zoo maintainers or workers taking care of cages in laboratories) showed less significant variations in the microbiota of workers. Nevertheless, these results, especially in the case of zoo maintainers, may have been influenced by the lack of investigation into the dietary habits and lifestyles of the participants and by the small sample size. In contrast to these results, in fact, a study has shown a similarity between the composition of the microbiota of monkeys and individuals who shared their environment and dietary habits [90]. Another category of workers exposed to biological risk studied was that of healthcare workers. In particular, hospital staff seems to be at greater risk of changes in the microbiota (mainly collected in skin samples), involving higher concentrations of pathogenic microorganisms in the tissues, if they work in specific departments (e.g., ICU) and if they have a long history of work experience at such departments. These results are consistent with what was previously reported regarding the influence of the hospital environment on hospitalized patients [91,92]. The studies on the microbiota present on the hands of healthcare workers reported that frequent hand washing is certainly a protective factor against infections, but if this practice involves huge alterations of the skin barrier and of the composition of the resident microbiota, then negative consequences for the individual health can occur. These findings are in line with recent studies showing how an alteration of the commensal microbiota of the hands can result in a lower defense or can even favor the invasion of pathogenic microorganisms in the skin and mucous membranes [93,94]. Regarding the biological risk, the results of our comprehensive literature review also suggested seasonal variability, therefore indicating dynamic and evolutionary features in the composition of the microbiota. Numerous pieces of evidence have already shown the dynamic effect that the seasons can have on the composition of the microbiota of children and adults [95,96]. In the occupational field, Kamuri et al. had further confirmed our hypotheses by demonstrating variability in the populations of bacteria and fungi found in environmental sampling of confined structures where workers were in close contact with pigs [97,98]. De Boeck et al., on the contrary, reported no correlation between microbial composition and seasonal variability in a study including healthy volunteers and not specifically workers exposed to direct contact with animals [99]. Our analysis suggests that these seasonal fluctuations could relate to occupational exposure to a certain risk factor (for example, biological) but not to others (for example, exposure to pesticides). In fact, in the only study concerning exposure to pesticides, changes in the microbiota of exposed workers were maintained throughout the year regardless of the passing of the seasons. Therefore, future studies investigating the changes in the workers’ microbiota according to different occupational factors and the seasonal variability are needed to better understand the interconnections in this relationship. Numerous studies confirm the modifications that pesticides can induce at the microbiota level and particularly involve the gut–brain axis [65,100,101]. Several studies have proven that there is a strong connection between the gut microbiota and the brain and called this connection “the gut–brain axis”. The evidence from animal and human studies has shown that gut microbiota can play an important role in brain behavior and cognitive development by producing hormones, immune factors, and metabolites, which also indicated that altering the gut microbiota may improve or even cure brain diseases [102]. The functions of the gut–brain axis are to coordinate gut functions and connect the emotional centers of the brain with the peripheral intestinal functions and mechanisms like an enteric reflex, intestinal permeability, immune activation, and enteroendocrine signaling [103]. It is essential to encourage the development of studies that investigate the occupational risk factor linked to pesticides since it is present in numerous occupational areas (e.g., agriculture). Our review also focused on analyzing the impact that the alteration of circadian rhythms deriving from specific forms of work organization (e.g., shiftwork and night work) can have on the microbiota. Recently, Ma et al. conducted an interesting experiment in which they simulated sleep deprivation (SD) and non-24-h work and rest hours (8 h on and 4 h off) and measured changes in a range of cognitive and microbiota parameters. Actigraphy data suggest that a 12 h schedule might result in chronic SD. Results of neurobehavioral psychomotor vigilance test and salivary biochemical parameters (including microbiota) demonstrated that an abnormal working and rest schedule might produce comprehensive interference with circadian rhythms, metabolism, and cognition [104]. Our review also focused on the possible impact of work-related stress on microbiota since the most recent literature underlines the close interconnection between stress and anxiety issues with microbiota modifications [105,106,107]. Work tasks characterized by huge physical and mental stress due to changes in the microclimate, long trips, and changes in dietary habits and lifestyle (e.g., tunnel workers, diving subsea, military, sailors) were studied. Our results showed in most cases that the persistence in stressful atmospheres and microclimates produced changes in the microbiota of workers. In only one case, these changes were associated with the development of stress/anxiety symptoms in tunnel workers, investigated through the use of questionnaires. In other cases, the correlation between long periods away from home and the development of transient pathologies such as traveler’s diarrhea was sought, thus seeking a correlation between the development of short-term health problems and changes in the microbiota. More studies focusing on the relationship between changes in the microbiota due to occupational exposure and the development of medium to long-term clinical conditions are needed. In our analysis, only two studies looked for a correlation between occupational diseases (in both cases of lung localization) and changes in the microbiota. Interesting correlations are emerging on the relationship between the lung microbiome and the development of diseases. Hence, it is very important to study this kind of disturbs also in the occupational field where respiratory diseases are widely spread [108]. Further developments could be fundamental to create ad hoc prevention and monitoring protocols for the protection of worker health in companies.

## 5. Conclusions

The objective of our review was to analyze how occupational exposure and types of work are factors that can influence the presence of different and specific microbiota. Occupational factors can interface with the biological rhythms of the bacteria and can contribute to the onset of diseases. The typing of the microbiota is essential to understand how the work environment can affect and modify the bacteria present in human organisms. In the future, the use of the microbiome as a biomarker can be a valid diagnostic and non-invasive monitoring support for an easily adaptable differentiation during Health Surveillance by Occupational Physicians to protect workers’ health and safety.

## Figures and Tables

**Table 1:** Tools for assessing the quality of studies included in this systematic review.

Scale	Examined Study	Questions	Scores Range
Insa	Narrative Reviews	N.7 (yes/no)	0–7 pt
New Castle Ottawa	Case-Control	Selection N.4, Comparability N.1, Exposure N.3 (yes/no)	0–8 pt
New Castle Ottawa	Cross-Sectional	Selection N.4, Comparability N.1, Outcome N.2 (yes/no)	0–10 pt
New Castle Ottawa	Cohort Studies	Selection N.4, Comparability N.1, Outcome N.3 (yes/no)	0–8 pt

**Table 2:** Studies included in the systematic review in alphabetical order.

Author	Year	Type of Study	Country	Score
Ahmed N. [39]	2019	Cross-sectional	Egypt	6
Grant E. [40]	2019	Cross-sectional	Thailand	5
Hang J. [41]	2017	Longitudinal	USA	4
Islam Z. [42]	2020	Longitudinal	Denmark	5
Kates AE. [43]	2019	Cross-sectional	USA	8
Khan F.M. [44]	2020	Narrative review	USA	5
Kraemer J.G. [45]	2019	Longitudinal	Switzerland	8
Lai P.S. [46]	2017	Cross-sectional	USA	4
Lai P.S. [47]	2019	Narrative review	USA	3
Lu ZH. [48]	2021	Longitudinal	China	7
Mbareche Z. [49]	2019	Case-control	Canada	7
Mortas H. [50]	2020	Cross-sectional	Turkey	5
Peng M. [51]	2020	Cross-sectional	USA	6
Reynolds A.C. [52]	2016	Commentary	Australia	1
Reynolds A.C. [53]	2016	Narrative review	Australia	4
Rocha L.A. [54]	2009	Case-control	Brazil	4
Rosenthal M. [55]	2014	Longitudinal	USA	6
Shukla SK. [56]	2017	Case-control	USA	6
Stanaway I.B. [57]	2016	Longitudinal	USA	7
Sun J. [58]	2017	Case-control	China	6
Sun J. [59]	2020	Longitudinal	China	7
Swanson G.R. [60]	2020	Cross-sectional	USA	7
Tan S.C. [61]	2020	Case-control	Malaysia	7
Walters W.A. [62]	2020	Longitudinal	Honduras	6
Wu BG. [63]	2020	Cross-sectional	USA	7
Wu J. [64]	2020	Cross-sectional	China	7
Yuan Y. [65]	2019	Cross-sectional	China	6
Zhang J. [66]	2020	Longitudinal	China	8
Zheng N. [67]	2020	Cohort	China	8
Zhou L. [68]	2019	Narrative review	China	5
Zhou Y. [69]	2019	Case-control	China	7

**Table 3:** Tools for microbiota samplings.

	Tot = 26
**Biological samples**	**26/26 (100%)**
Fecal sample	10/26 (38.4%)
Nasal swab	9/26 (34.6%)
Oral swab	6/26 (23%),
Skin sample	5/26 (19.2%)
Blood sample	3/26 (11.5%)
Nasopharyngeal swabs	2/26 (7.7%)
Glove juice	1/26 (3.8%)
**Environmental samples**	**7/26 (27%)**
Air sample	7/7 (100%)
Fluid sample	3/7 (42.8%)

**Table 4:** Occupational exposure and workers’ categories.

	Tot = 26
**Exposure to biological agents**	**15/26 (57.7%)**
Work with animals	12/15 (80%)
*Farmers and slaughters*	10/12 (83.4%)
*Zookeepers*	1/12 (8.3%)
*Lab personnel*	1/12 (8.3%)
Healthcare workers	3/15 (20%)
**Shift workers**	**2/26 (7.7%)**
**Exposure to chemical agents**	**4/26 (15.3%)**
Metalworking fluid	1/4 (25%)
Pesticides	1/4 (25%)
Dust (ceramic, silica)	2/4 (50%)
**Exposure to stress factors and microclimate**	**5/26 (19.3%)**
Military	2/5 (40%)
Sailors	1/5 (20%)
Diving sub-sea	1/5 (20%)
Tunnel workers	1/5 (20%)

## Supplementary Materials

The following are available as Supplementary Material, online at https://www.mdpi.com/1660-4601/19/3/1043/s1, Table S1: Summary of contents of the case-control and cohort studies included in the review, Table S2: Summary of contents of the cross-sectional articles included in the review, Table S3: Summary of contents of the review articles included in the review.
