# Correction: Mucci et al. WORKbiota: A Systematic Review about the Effects of Occupational Exposure on Microbiota and Workers’ Health. *Int. J. Environ. Res. Public Health* 2022, *19*, 1043

**DOI:** 10.3390/ijerph192013678

**Published:** 2022-10-21

**Authors:** Nicola Mucci, Eleonora Tommasi, Annarita Chiarelli, Lucrezia Ginevra Lulli, Veronica Traversini, Raymond Paul Galea, Giulio Arcangeli

**Affiliations:** 1 Department of Experimental and Clinical Medicine, University of Florence, 50139 Florence, Italy; 2 Postgraduate Medical Training Programme in Cardiology, University of Perugia, 1 Piazza dell’Università, 06123 Perugia, Italy; 3 Occupational Medicine Unit, Careggi University Hospital, 50134 Florence, Italy; 4 Occupational Medicine School, University of Florence, 50139 Florence, Italy; 5 Faculty of Medicine & Surgery, University of Malta, MSD 2090 Msida, Malta; 6 The Malta Postgraduate Medical Training Programme, Mater Dei Hospital Msida, MSD 2090 Msida, Malta

## Addition of an Author

Eleonora Tommasi was not included as an author in the original publication [1]. The corrected Author Contributions Statement appears here. The authors apologize for any inconvenience caused and state that the scientific conclusions are unaffected. The original publication has also been updated.
