# Supplementary material for: WORKbiota: A Systematic Review about the Effects of Occupational Exposure on Microbiota and Workers’ Health
Source: Int J Environ Res Public Health. 2022 Jan 18;19(3):1043. doi: 10.3390/ijerph19031043 (PMC8834335; doi:10.3390/ijerph19031043)
Supplement: Supplementary file 1 [file ijerph-19-01043-s001.zip › ijerph-1464520-supplementary/Table S1.pdf]

Table S1. Summary of contents of the case-control and cohort studies included in the review.

Author	Number Participants	Population	Outcome	Tools	Results
Hang J. [38]	50	military personnel	characterize the upper respiratory microbiomes of healthy military personnel in a garrison environment	nasal, oro-pharyngeal, and nasopharyngeal swabs	Staphylococcus, Corynebacterium, and Propionibacterium were more than 75% of all OTUs in nasal and nasopharyngeal microbiota. Streptococcus was dominant (approximately 50% of all OTUs) in the oropharynx.
Islam Z. [39]	28	pig farmworkers	investigate the composition and temporal dynamics of the nasal microbiome in people during and after long- and short-term exposure to LA-MRSA CC398-positive pig farms	questionnaires and nasal swab	PCR analysis of 221 pig farmworkers samples showed that positivity for LA-MRSA CC398. All 16 samples collected immediately after the visits were previously shown to be positive for MRSA, whereas all 32 samples collected 2 hours before or 48 hours after the visits were MRSA-negative.
Kraemer J.G. [42]	101	animal farmers	demonstrate an influence of seasons on the similarity between nasal microbiota of animal farmers and air barns' composition	nasal swab. Enviromental samples	There were significant differences between winter and the remaining three seasons in pig farmers (P 0.01 [spring],P 0.001 [summer], and P 0.03 [fall] for SDI; P 0.001). The nonexposed group had consistent and low richness and SDI values with no differences between seasons (P 0.05 for richness; P 0.05 for SDI).
Lu ZH. [45]	48	tunnel workers	Assessing correlation between changes in the microbiome and mental state in workers in a tunnel environment	questionnaire, fecal samples	Gut microbial diversity was lower after tunnel exposure [Shannon ($t = 3.375$, $P = 0.001$) and Simpson ($t = 2.757$, $P = 0.008$) indices]. A higher abundance was found in the phylum Actinobacteria ($LDA = 4.50$, $P = 0.001$). The self-evaluation showed that at least one-half of the tunnel workers experienced one or more symptoms of mental distress.
Mbareche Z. [46]	54	swine workers	evaluate the correlation between nasopharyngeal bacterial flora of swine workers and the swine barns bioaerosol biodiversity.	air samples and nasopharyngeal samples	In farmers, Firmicutes and Bacteriotedes were the most dominant phyla with relative abundances of 40%, and 24%, respectively. Air samples from pig farms had comparable profiles to the nasopharyngeal flora of farmers, with a dominance of Firmicutes (83%), followed by Bacteriotedes (11%). The clustering (air from pig buildings and pig farmers together, versus the non-exposed controls) was statistically significant as confirmed by the PERMANOVA test (p-value = 0.0001).

Rocha L.A. [51]	60	nurses	evaluate the microbial flora of nurses' healthy and damaged hand by frequent handwashing and/or wearing of gloves.	Hand Skin Assessment Form, hand skin swabs	Damaged hands had higher frequency of Staphylococcus aureus, 16.7% versus 10%; gram-negative bacteria, 20% versus 6.7%; and yeast, 26.7% versus 20%. Staphylococcus haemolyticus was only in damaged hands (P 5 .02). MRSA and gram-negative bacteria was also greater among damaged hands.
Rosenthal M. [52]	34	nurses	examined the association among hand microbial community structure, potential pathogen carriage, and hand hygiene practices	Visual Scoring of Skin Scale, Hand Skin Assessment Scale, swab and glove-juice samples	S. aureus ranged from 41.2% to 52.9%; Enterococcus spp. ranged from 52.9% to 61.8%; C. albicans ranged from 2.9% to 8.8% and MRSA ranged from 2.9% to 5.9%. S. aureus and Enterococcus spp. co-occurred the most frequently, ranging from 29.4% to 35.3% across collection visits.
Shukla SK. [53]	39	livestock farmers	investigate differences between microbiota of dairy farmers (DF) compared with urban non-farmers (NF)	nasal and oral swab	(N_DF) showed maximum species richness compared with that of the other three sample groups: N_NF, O_DF, O_NF. Specifically, the Chao 1 richness was significantly higher in N_DF when compared to N_NF (p-value = 1.55E-07), to O_NF (p-value = 1.24E-07) and to O_DF (p-value = 1.03E-05).
Stanaway I.B. [54]	117	farmworkers	investigate agricultural pesticide exposure-associated changes in the oral buccal microbiota	oral and blood samples	Azinphos-methyl was detected in 36 farmworkers. Streptococcus, Micrococcineae, Gemella, Haemophilus, Halomonas, Actinomycineae, and Granulicatella were significantly reduced in the spring/summer in azinphos-methyl group. Mannheimia, Herminiimonas, Actinobacillus, Neisseria, and Prevotella appear also to be suggestively perturbed in the spring/summer in azinphos-methyl group.
Sun J. [55]	12	pig farmers	compare fecal microbial composition and antibiotic resistant genes between farm workers, local villagers and swine.	fecal samples	Farm workers and swine had significantly higher and lower relative abundances of Bacteroidetes and Firmicutes. Clostridiaceae were more abundant in the feces of farm workers and swine compared with the local villagers. Enterobacteriaceae and Lachnospiraceae in the workers feces were more abundance than those from the local villagers.
Sun J. [56]	14	veterinary students	study the impact of confined and controlled swine farm environments on temporal changes in the gut microbiome and resistome of veterinary students with	fecal samples, enviromental samples	Multivariate analysis of operational taxonomic unit (OTU) composition revealed a modest yet significant change ($R2$=7.4%, permutational multivariate analysis of variance [PERMANOVA] P<0.001). Specific draft genomes, rarely observed in the human gut, shared 99.9 ± 0.1% (minimum 99.7%) 16S rRNA gene similarity and 99.5 ± 0.4% (minimum 98.9%) average nucleotide identity (ANI) between exposed students' gut microbioma and enviromental samples

			occupational exposure for 3 months.		
Tan S.C. [58]	32	pig farmers	evaluate the effect of close human-animal interaction to the faecal metagenome and metabonome of swine, farmer and human control.	fecal samples	Bacteroidetes, Firmicutes and Proteobacteria made up >93% of the phyla detected in the faecal samples from all groups. Farmer and swine harboured higher relative abundance of Firmicutes (29% in swine, 27% in farmers and 15% in human control) and Proteobacteria (12% in swine, 8% in farmers and 4% in human control) than human control.
Walters W.A. [59]	67	military personnel	study the interactions between the gut microbiome and Traveler Diarrhea	fecal samples and questionnaire	Ruminococcaceae UCG-013 were found in TD+ subjects while Ruminiclostridium sp. had higher relative abundance in TD- subjects. Haemophilus (p-value 0.0007) and Turicibacter sp (p value 0.016) were shown to have a positive relationship with GI distress.
Zhang J. [63]	82	sailors	explore the impacts of sea voyage on the intestinal microbiome of sailors	questionnaire and fecal samples	After sea voyage sailor had an increase of Streptococcus gordonii and Klebsiella pneumoniae in fecal samples
Zheng N. [64]	255	healthcare workers	evaluate the alteration of the gut microbiome in medical workers	fecal samples, enviromental samples	Firmicutes was more abundant in the ST (average relative abundance, 66.5%; $q = 0.002$) and LT (59.7%; 199 $q = 0.033$) workers than in the NCs (55.8%), while Bacteroidetes was less abundant in workers (average relative abundance in ST, LT and NC groups, 22.1% [$q = 0.00016$], 27.2% [$q = 0.009$] and 33.6%, respectively).
Zhou Y. [66]	39	silica-exposed workers	clarify characteristics of the gut microbiota in early-stage silicosis and understand the influence on pulmonary fibrosis.	blood and fecal samples	Firmicutes and Actinobacteria were significantly lower in patients with silicosis than in healthy subjects ($p < 0.05$);Proteobacteria, Lentisphaerae, Tenericutes and Cyanobacteria were higher ($p < 0.05$). Blautia and Pseudomonas aeruginosa were decreased in the fecal samples of the patients, Megamonas,Dialiste and Ruminiclostridum were significantly increased
