# Supplementary material for: WORKbiota: A Systematic Review about the Effects of Occupational Exposure on Microbiota and Workers’ Health
Source: Int J Environ Res Public Health. 2022 Jan 18;19(3):1043. doi: 10.3390/ijerph19031043 (PMC8834335; doi:10.3390/ijerph19031043)
Supplement: Supplementary file 1 [file ijerph-19-01043-s001.zip › ijerph-1464520-supplementary/Table S2.pdf]

Table S2. Summary of contents of the cross-sectional articles included in the review.

Author	Number of Participants	Population	Outcome	Tools	Results
Ahmed N. [36]	19	ceramic factory workers	analysing differences in the nasal microbiota between workers in the ceramics industry	nasal swabs	Rural samples were found to have a higher significant increase in the representation of Actinobacteria (p = 0.004), whereas the industrial group of samples displayed a significant increase in the relative abundance of phylum Proteobacteria (p = 0.002). The higher significant relative abundance of Bacteroidetes was observed in rural samples (p = 0.01).
Grant E. [37]	18	zootechnical workers	possible influence of macaque microbiota on zoo workers' microbiota	questionnaire and fecal sample	All sample profiles were dominated by Bacteroidetes, Firmicutes, and Proteobacteria. Alpha diversity was significantly lower among human exposed relative to human controls based on the observed features (p = 0.04), Shannon's index (p = 0.02), and Simpson's index (p = 0.04).
Kates AE. [40]	59	livestock farmers	assess the microbial composition of the anterior nares and oropharynx of livestock workers	nasal and oropharynx swabs and questionnaire	24 OTUs were significantly more abundant in those with livestock contact. In those over 55, individuals with livestock exposure Streptococcus genus was the most prevalent genus observed in the oropharynx followed by Prevotella and Haemophilus genera. When comparing swine workers to those with any other animal contact, one OTU was significantly more abundant in the swine workers, Clostridiumsensustricto (Log 2 -fold change: 8.58, P < 0.001).
Lai P.S. [43]	10	animal care workers	the effect of environmental microbial exposures on the human microbiome in animal care workers.	nasal, oral, and skin samples. Environmental samples	The average proportion of pre-shift microbiome was: 0.1 ± 0.1% for the oral microbiome; 3.1 ± 1.9% for the nasal microbiome; 3.0 ± 1.5% for the skin microbiome. The average proportion of post-shift microbiome was: 0 ± 0% for the oral microbiome; 3.7 ± 2.1% for the nasal microbiome; 14.1 ± 28.5% for the skin microbiome.There were no significant differences in community structure of the environmental microbiome in the dirty cage wash areas as measured by area samplers vs. personal samplers worn by the participants
Mortas H. [47]	10	shiftworkers	determine the differences in the gut microbiota of rotational shift workers	fecal sample	Night-shift workers: an increase of Firmicutes and Actinobacteria, Bacteroidetes decreased. Dorea longicatena and Dorea formicigenerans were more abundant after night-shift (p = 0.005). Faecalibacterium was found to be a biomarker of the day shift work.

Peng M. [48]	30	farmworkers	investigate the environmental influences introduced by livestock/poultry operations on forearm skin microflora of on-site farm workers	skin swab	On forearm skin of farmworkers were found significantyly higher levels of Proteobacteria (Pseudomonas and Acinetobacter, $p < 0.05$) and lower of Actinobacteria (Corynebacterium and Propionibacterium, $p < 0.05$) respect to controls
Swanson G.R. [57]	42	shiftworkers	Analyze the impact of night shift work on microbiota derived short chain fatty acids (SCFAs)	questionnaire and blood samples	SCFA had diurnal oscillation: acetate ($F = 4.5$, $P < 0.01$), propionate ($F = 2.9$, $P < 0.01$), Butyrate/Total ($F = 3.1$, $P < 0.01$), and Total SCFA ($F = 4.6$, $P < 0.01$). SCFA in NW subjects lost 24-hour rhythmicity.
Wu BG. [60]	302	metalworking fluid workers (MWF)	evaluate whether exposure to microbes in MWF from the plant influenced workers' respiratory tract microbiota and potentially stimulated a local B cell response	lung biopsies, skin wabs, environmental samples	Results from patologic lung biopsies of exposed workers were: bronchiolocentric lymphoplasmacytic infiltrates with CD20-positive B-cell primary lymphoid follicles without germinal centers, involving both bronchioles and alveolar ducts; scattered CD3-positive T-cells predominantly cuffing the B-cell follicles; no appreciable interstitial or airway fibrosis nor granulomas; and airspace enlargement with septal wall fragmentation, indicative of mild to moderate histological emphysema. An OTU annotated to Pseudomonas (Pseudomonas_813945) was differentially enriched in lung, skin, and nasal samples as well as MWF (P. andersonii, P. mendocina, P. pseudoalcaligenes and P. oleovorans).
Wu J. [61]	12	animal workers	compare the microbiota in different pig-present settings	nasal samples and air sample	Bacterial richness of pig farmer's nasal samples was higher than slaughter nasal samples; pig farm bioaerosol samples had higher abundances than slaughter bioaerosol ones ($p < 0.05$ or $p < 0.01$). There were 31.7% shared OTUs between pig farm bioaerosols and pig farmers which was higher than that between pig slaughterhouses and slaughterhouse workers (23.4%) ($p < 0.001$).
Yuan Y. [62]	47	diving subsea workers	influence of commercial helium–oxygen saturation diving on divers' gut microbiotas	faecal samples	Bifidobacterium and (scFa)-producing bacteria (Fusicatenibacter, Faecalibacterium, and Anaerostipes) decreased before, during and after diving. Lactococcus garvieae, Actinomyces odontolyticus, Peptoclostridium difficile, Butyricimonas virosa, Streptococcus mutans, Porphyromonas asaccharolytica and A. graevenitzii (most of them pathogens) increased.
