# Supplementary material for: WORKbiota: A Systematic Review about the Effects of Occupational Exposure on Microbiota and Workers’ Health
Source: Int J Environ Res Public Health. 2022 Jan 18;19(3):1043. doi: 10.3390/ijerph19031043 (PMC8834335; doi:10.3390/ijerph19031043)
Supplement: Supplementary file 1 [file ijerph-19-01043-s001.zip › ijerph-1464520-supplementary/Table S3.pdf]

Table S3. Summary of contents of the review articles included in the review.

Title	Author	Year	Type of Study	Results
Environmental exposures and Autoimmune Diseases: Contribution of Gut Microbiome [41]	Khan F.M.	2020	Narrative review	Trichloroethene (TCE) exposure, which is known to induce/exacerbate systemic lupus erythematosus in both experimental animals and humans, is also reported to cause alterations in the gut microbiome with increased abundance of genus Bifidobacterium and bacterial family Enterobacteriaceae as well as lower abundance of the genus Bacteroides and Lactobacillus in MRL +/+ mice at a high but occupationally relevant TCE dose compared to controls.
"Gut Microbiota-Circadian Clock Axis" in Deciphering the Mechanism Linking Early-Life Nutritional Environment and Abnormal Glucose Metabolism [65]	Zhou L.	2019	Narrative review	If the rhythmic feeding times are disrupted, such as host genetic molecular clock deficiency andt ime-shift-inducedjetlag, then aberrant gut microbiota diurnal rhythmicity and dysbiosis occurred. several experimental animal models indicated that there was bidirectional communication between gut microbiota and circadian clock and the changed microbial metabolites could further influence the circadian clock and metabolic health.
Impact of occupational exposure on human microbiota [44]	Lai P.S.	2019	Narrative review	Recent evidence suggests that work-related microbial and nonmicrobial exposures change the adult human microbiome. Work with animals appears to be associated with increased microbial diversity in the nasal microbiome of adult pig and dairy farmers. There is growing recognition that environmental chemical, metal and particle exposures can change the human microbiome. In both human studies and animal models, arsenic at environmentally relevant concentrations alters the gut microbiome.
Sleepy, circadian disrupted and sick: Could intestinal microbiota play an important role in shift worker health? [49]	Reynolds A.C.	2016	Commentary	More recent research has shown that sleep and circadian disruption, via clock gene mutation or weekly shifts of the light-dark cycle, can negatively impact gastrointestinal tract function and produce dysbiosis, especially when combined with alcohol induced colitis or a high-fat high-sugar diet
The shift work and health research agenda: Considering changes in gut microbiota as a pathway linking shift work, sleep loss and circadian misalignment, and metabolic disease [50]	Reynolds A.C.	2016	Narrative review	Both physiological and psychological stress are consistently linked with disruption to the gut microbiota, and increased gut permeability. Given that sleep loss and circadian misalignment are considered physiological stressors, a stressor-gut microbiota-inflammation-metabolic function pathway may explain the relationship between shift work, obesity, and obesity-related disease.
